# CBA (4-chloro-2-(2-chlorophenoxy)acetamido) benzoic acid) inhibits TMEM206 mediated currents and TMEM206 does not contribute to acid-induced cell death in colorectal cancer cells

**DOI:** 10.3389/fphar.2024.1369513

**Published:** 2024-03-07

**Authors:** Sven Kappel, Korollus Melek, Daniela Ross-Kaschitza, Barbara Hauert, Christian E. Gerber, Martin Lochner, Christine Peinelt

**Affiliations:** Institute of Biochemistry and Molecular Medicine, University of Bern, Bern, Switzerland

**Keywords:** TMEM206, PAORAC, ASOR, small molecule inhibitor, colorectal cancer

## Abstract

**Introduction:** Upon activation at low pH, TMEM206 conducts Cl^−^ ions across plasma and vesicular membranes. In a (patho)physiological context, TMEM206 was reported to contribute to acid-induced cell death in neurons, kidney and cervical epithelial cells. We investigated the role of TMEM206 in acid-induced cell death in colorectal cancer cells. In addition, we studied CBA as a new small molecule inhibitor for TMEM206.

**Methods:** The role of TMEM206 in acid-induced cell death was studied with CRISPR/Cas9-mediated knockout and FACS analysis. The pharmacology of TMEM206 was determined with the patch clamp technique.

**Results:** In colorectal cancer cells, TMEM206 is not a critical mediator of acid-induced cell death. CBA is a small molecule inhibitor of TMEM206 (IC_50_ = 9.55 µM) at low pH, at pH 6.0 inhibition is limited.

**Conclusion:** CBA demonstrates effective and specific inhibition of TMEM206; however, its inhibitory efficacy is limited at pH 6.0. Despite this limitation, CBA is a potent inhibitor for functional studies at pH 4.5 and may be a promising scaffold for the development of future TMEM206 inhibitors.

## 1 Introduction

The most abundant anion in the human body is chloride (Cl^−^). It regulates many physiological functions, i.e., cell volume, vesicular acidification, transepithelial transport, and cellular signaling (Jentsch et al., 2002; Jentsch and Pusch, 2018). Like other charged molecules, anions cannot freely cross the plasma membrane and rely on active and passive transport mechanisms. Several cellular signals can activate Cl^−^ channels. Phosphorylation-dependent and independent mechanisms activate the cystic fibrosis transmembrane conductance regulator (CFTR) (Moran, 2017) and intracellular calcium ions (Ca^2+^) activate anoctamin-1 (ANO-1, TMEM16A) (Caputo et al., 2008). Current research focusses on Cl^−^ channels that are activated by increased extracellular proton (H^+^) concentrations. So-called proton-activated outwardly rectifying anion channels (PAORAC) or acid-sensitive outwardly rectifying (ASOR) channels mediate Cl^−^ flux upon extracellular acidification (Lambert and Oberwinkler, 2005; Wang et al., 2007; Ma et al., 2008). TMEM206, the molecular component underlying PAORAC/ASOR, has been identified by two independent research groups in 2019 (Ullrich et al., 2019; Yang et al., 2019). Also, the structure of TMEM206 has been resolved recently: TMEM206 forms a homo-trimeric channel with each monomer having two transmembrane-spanning helices (Ruan et al., 2020; Deng et al., 2021). According to the human protein atlas, TMEM206 shows an almost ubiquitous mRNA expression with the most prominent expression in the brain, kidneys, and lymphoid tissues (The Human Protein Atlas, 2023). Yet its biological function has not yet been fully understood. On a sub-cellular level, Cl^−^ conductance by TMEM206 was reported to prevent endosomal hyperacidification (Osei-Owusu et al., 2021). In addition, TMEM206 has been found to contribute to the shrinkage of macropinosomes, a special type of endosomes with particular importance in immune and cancer cells. Disruption of TMEM206 reduces macropinosome resolution and increases albumin-dependent survival of cancer cells (Zeziulia et al., 2022). In addition to its abundance in vesicles, TMEM206 also localizes to the plasma membrane.

In the plasma membrane, TMEM206 was reported to contribute to acid-induced cell death in cultured neuronal cells from mice, HeLa, and HEK293 cells (Wang et al., 2007; Sato-Numata et al., 2014; Ullrich et al., 2019). Wang et al. proposed that TMEM206 plays a role in pathologies like ischemic stroke and cancer where pH might drop below 6.5 (Xiong et al., 2004; Kato et al., 2013; Thews and Riemann, 2019). Although the activation threshold is below pH 5.5 at room temperature it shifts to ∼ pH 6.0 at 37°C (Sato-Numata et al., 2013) so TMEM206 might be activated in pathophysiological conditions. Certain compartments within the human body also show pH values close to the TMEM206 activation threshold. In the colon, pH values range from pH 5.7 in the caecum that slowly increases to pH 6.7 in the rectum (Fallingborg, 1999), thus TMEM206 might also play a role in colonic epithelium in general and colorectal cancer, where pH might be even lower than under physiological conditions. Therefore, we were wondering if TMEM206 is expressed in human colorectal cancer cells and if it contributes to acid-induced cell death.

To better understand the contribution of TMEM206 to cellular functions, there is a need for pharmacological tools. Pharmacological inhibition of the channel avoids compensatory mechanisms that follow knockout or knockdown. TMEM206 is inhibited by the common Cl^−^ channel inhibitor DIDS (4,4′-diisothiocyano-2,2′-stilbenedisulfonic acid), flufenamic acid, and the anti-rheumatic niflumic acid (Lambert and Oberwinkler, 2005; Capurro et al., 2015) but those compounds are not specific for TMEM206 (Liantonio et al., 2007; Guinamard et al., 2013). In addition, phloretin (Wang et al., 2007) and pregnenolone sulphate (PS) (Drews et al., 2014) have been reported as PAORAC/ASOR/TMEM206 inhibitors, however, phloretin is an inhibitor of the sodium glucose cotransporter (SGLT) (Habtemariam, 2023) and pregnenolone sulphate an activator of TRPM3 (Wagner et al., 2008). A recent study identified phosphatidylinositol-4,5-bisphosphate (PIP_2_) as a novel inhibitor of TMEM206 (Mihaljević et al., 2023). But, PIP_2_ has also been reported as a modulator of a diverse set of ion channels (Suh and Hille, 2008; Gada and Logothetis, 2022), including the Cl^−^ channels TMEM16A and TMEM16B (Ta et al., 2017). Taken together, there is a need for specific TMEM206 inhibitors or at least small molecules, that serve as a scaffold for selective and more potent inhibitors.

Lately, ligand-based virtual screening (LBVS) with 3D-shape and pharmacophore similarity algorithms xLOS (atom category eXtended Ligand Overlap Score) has advanced the discovery of ion channel inhibitors (Simonin et al., 2015). When, amongst other known weak and unspecific inhibitors, flufenamic acid is given as inhibitor template, LBVS identified 4-chloro-2-(2-chlorophenoxy)acetamido) benzoic acid (CBA), 4-chloro-2-(1-naphthyloxyacetamido) benzoic acid (NBA) and 4-chloro-2-(2-(4-chloro-2-methylphenoxy)propanamido) benzoic acid (LBA) as potential inhibitors (Delalande et al., 2019). In fact, flufenamic acid inhibits transient potential melatonin 4 (TRPM4) ion channel and CBA, NBA and LBA could be identified as potent small molecule inhibitors of TRPM4 (Ozhathil et al., 2018; Borgström et al., 2021a; 2021b; Stokłosa et al., 2021; Preti et al., 2022). CBA has been identified as specific inhibitor of TRPM4. In fact, CBA does not affect other members of the TRP channel family (TRPM5, TRPM7, TRPM8, TRPV1, TRPV6) or 17 other ion channel targets including GABA_A_ receptor α1 subunit, NMDA receptor, Ca^2+^ channels and voltage-gated K^+^ channels, which are targets of the parent drug flufenamic acid (Ozhathil et al., 2018).

In this study, we set out to test the contribution of TMEM206 to acid-induced cell death in the colorectal cancer cells. Additionally, we investigated CBA, NBA and LBA as inhibitors of TMEM206-mediated currents.

## 2 Materials and methods

### 2.1 Cell culture

All cells were cultivated at 37°C in humidified air with 5% CO_2_ in McCoy’s 5A medium supplemented with 10% FCS.

### 2.2 siRNA-mediated knockdown

To mediate TMEM206 knockdown, cells have been transfected with the Flexitube gene solution GS55248 (QIAGEN). Cells were transfected in a 4D Nucleofector system (Lonza) according to the manufacturer’s instructions and the recommended transfection solution for HCT116 cells.

### 2.3 CRISPR/Cas9-mediated knockout

With the help of the CRISPR/Cas9 system, TMEM206-knockout cell lines were generated. Guide RNAs were designed with the web tool CRISPOR (Concordet and Haeussler, 2018). By using two guide RNAS (gRNAs), 67 to 68 base pairs were deleted in exon 1 (Chr 1: 212′414′732 to 212′414′798 or 212′414′731 to 212′414′798). The oligonucleotides for the gRNAs were cloned into the pSpCas9(BB)-2A-GFP (PX458) (Addgene #48138) vector, according to the protocol from Addgene (Addgene, 2023). The transfection of the cells was performed as described in the transfection section with 2 µg of each gRNA plasmid (gRNA92rv/gRNA170fw). 24 h later cells were sorted in the ASTRIOS flow cytometry sorter for GFP positive cells. Single cells were seeded in four 96-wellplates. Approximately 2 weeks later, single cell clones could be transferred to 24-well plates. Around 100 clones were taken as single clones from the 96-well plates. The screening of the clones was done by genotyping. Clones showing the desired deletion in both alleles were amplified and then stocked in liquid N_2_. Cell pellets were taken, and qPCR was done for further validation. The deletion was confirmed by Sanger sequencing.

### 2.4 Electrophysiology

All experiments were performed in whole-cell configuration at room temperature. Voltage ramps spanning from −150 mV to +150 mV for TMEM206 and from -100 mV to +100 mV for TRPM4 with a duration of 50 ms were applied every 2 s. Induced currents were acquired with an EPC-10 amplifier (HEKA), recorded and digitized with Patchmaster (v2x53, HEKA). Voltages were corrected for a liquid junction potential of 10 mV. Currents were filtered at 1 kHz and then sampled at 3 kHz. To evaluate current development, currents were extracted at −130 and +130 mV for TMEM206 and at −80 and +80 mV for TRPM4, normalized to the cell capacitance, and plotted *versus* time. To further analyze data IgorPro 6.37 (Wavemetrics) and GraphPad Prism 9 were used. Bath solutions contained: 140 mM NaCl, 3 mM MgCl_2_, 0.5 mM CaCl_2_, 10 mM HEPES and pH was adjusted to 7.2 with HCl. In the pH 4.5 bath solution, 10 mM HEPES was replaced by 5 mM Na_3_-citrate and pH was adjusted to 4.5 with citric acid. Pipette solution contained: 120 mM Cs-glutamate, 10 mM HEPES, 3 mM MgCl_2,_ 20 mM Cs-BAPTA and pH was adjusted to 7.2 with CsOH (for TMEM206) and 140 mM Cs-glutamate, 10 mM EDTA, 10 mM HEPES, 8 mM NaCl, and final concentrations of 3 mM MgCl_2_ and 10 µM CaCl_2_ (for TRPM4 according to (Kappel et al., 2019)). If necessary, Osmolarity was adjusted to ∼310 mOsm with glucose.

### 2.5 Acid-induced cell death

Acid-induced cell death of HCT116 and HCT116 TMEM206 KO cells (clone 1-6) was assessed by staining with caspase 3/7 and SYTOX AADvanced Dead Cell stain. HCT116 and KO cells were incubated for 2.5 h at 37°C with DMEM medium at pH 4.5. For the quantification of caspase-3/7 activity, cells were stained with CellEvent caspase-3/7 green flow cytometry assay kit (ThermoFisher Scientific) according to the manufacturer’s protocol. Caspase activity was measured by flow cytometer using the BD LSR II SORP flow cytometer and analyzed using Flowjo software.

### 2.6 Quantitative PCR

Total RNA from HCT116 cells was isolated with QIAshredder and RNAeasy kit (both QIAGEN). RNA was reversely transcribed with the High-Capacity cDNA Reverse Transcription kit (Thermo Fisher). For the TMEM206 TaqMan Gene Expression Assay, a 1:4 dilution of cDNA was used. The PCR conditions in the CFX system (Bio-Rad) were: 2-min activation at 50°C, then 10 min at 95°C; followed by 40 cycles of 15-s denaturation at 95°C and 1-min annealing at 60°C. TMEM206 expression was normalized to TATA box-binding protein and RNA-polymerase II expression.

## 3 Results

### 3.1 TMEM206 is functionally expressed in colorectal cancer cell lines

**FIGURE 1:**
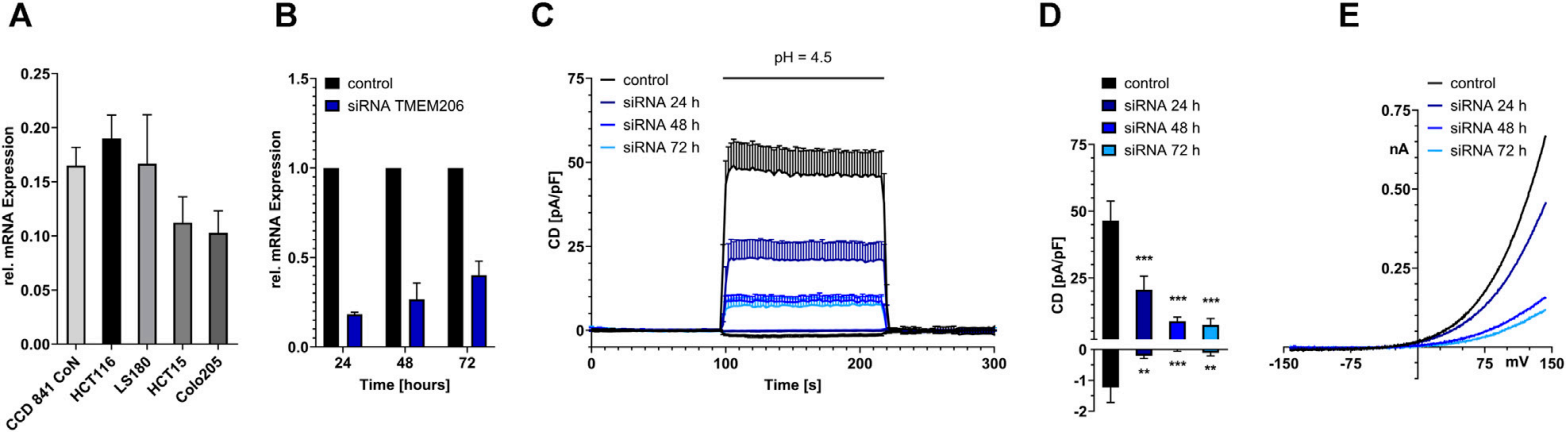
Functional expression of TMEM206 in colorectal cells. **(A)** Relative TMEM206 mRNA expression in different colorectal cancer cell lines and the non-cancerous cell line CCD 841 CoN. **(B)** TMEM206 mRNA expression after siRNA mediated knockdown in HCT116 cells (n = 3). **(C)** TMEM206 mediated currents 24 h (n = 13), 48 h (n = 14), and 72 h (n = 12) post siRNA knockdown and non-silencing control in HCT116 (n = 9). **(D)** Corresponding current densities extracted from **(C)** at t = 208 s **(E)** Corresponding current-voltage relationships to **(C)**. Statistical significance was determined by an ordinary one-way ANOVA.

Although database research already revealed that TMEM206 mRNA should be expressed in colonic tissue we performed qPCR to compare TMEM206 expression in four colorectal cancer cell lines with different dukes’ staging (HCT116, Dukes’ stage A, LS180, Dukes’ stage B, HCT15, Dukes’ stage C, Colo205, Dukes’ stage D) to the expression in the non-cancerous cell line CCD841 CoN. TMEM206 mRNA is expressed in all the tested cell lines with the highest expression in HCT116, similar expression levels in CCD841 CoN and LS180, and slightly lower expression in HCT15 and Colo205 that represent more advanced cancer stages (Figure 1A). Because HCT116 had the highest mRNA expression we used those cells for our experiments.

To test if TMEM206 is functionally expressed at the protein level in HCT116, we performed siRNA-mediated knockdown targeted at TMEM206. TMEM206 mRNA expression is reduced to 18% of the expression of the control transfected cells 24 h post transfection. The expression slightly increases to 26% and 40% after 48 and 72 h, respectively (Figure 1B). To determine if the reduction in mRNA expression is also reducing TMEM206 protein expression, whole-cell patch clamp experiments have been performed. In control-transfected HCT116 cells, pH 4.5 induced outward currents are at ∼46 pA/pF. Currents are reduced to ∼20 pA/pF after 24 h and are further decreased to ∼9 pA/pF and ∼7 pA/pF at 48 and 72 h after transfection, respectively (Figures 1C, D). Inward currents are reduced to a similar extent. The current-voltage relationships (Figure 1E) reveal a strong outward rectification reminiscent of TMEM206 currents.

### 3.2 TMEM206 does not contribute to acid-induced cell death in HCT116 cells

**FIGURE 2:**
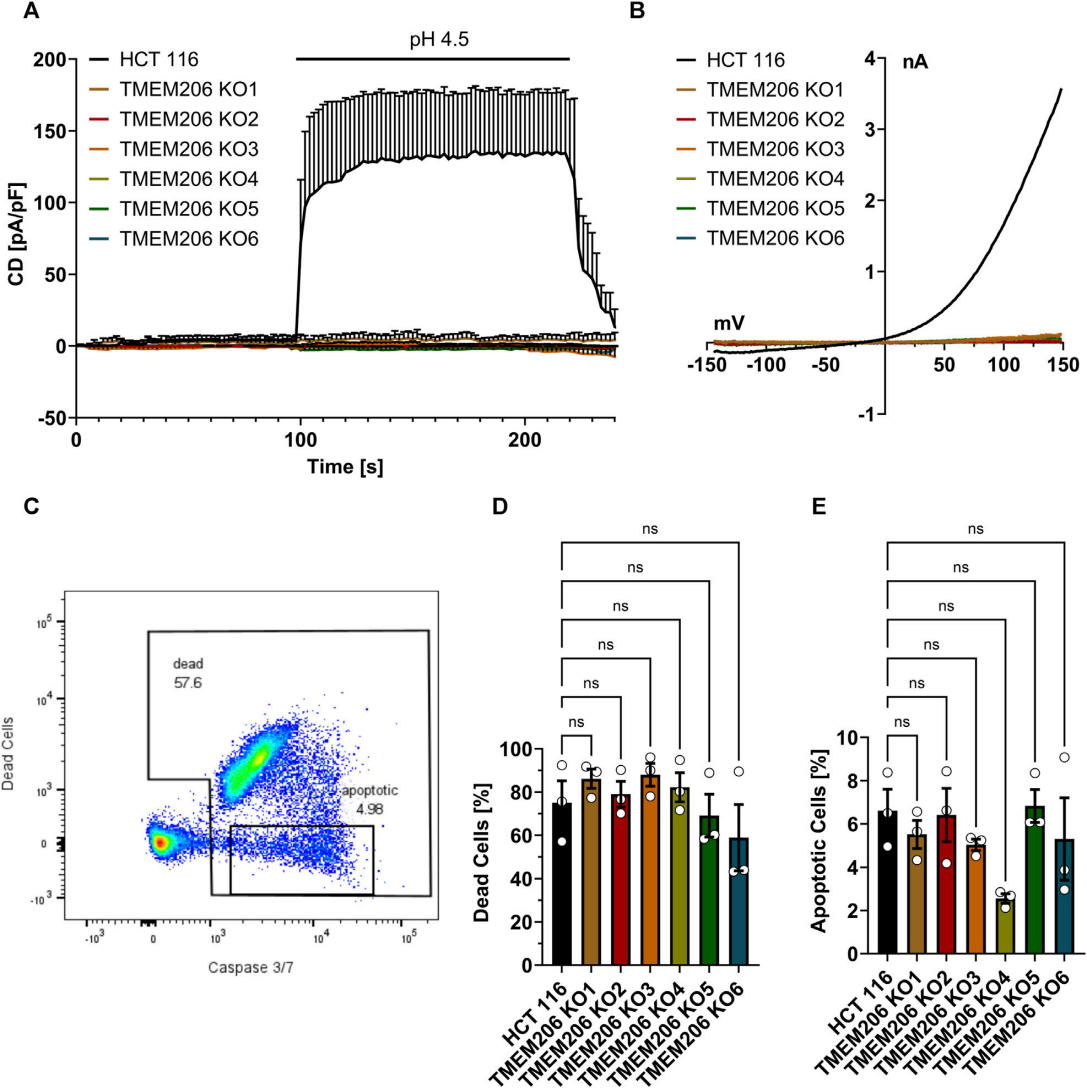
TMEM206 KO verification and acid-induced cell death in TMEM206 KO cells. **(A)** Current densities when low pH was applied as indicated in HCT116 vs TMEM206 KO cells (HCT116, n = 4; TMEM206 KO1, n = 3; TMEM206 KO2, n = 2; TMEM206 KO2, n = 3; TMEM206 KO4, n = 3; TMEM206 KO5, n = 2; TMEM206 KO6, n = 1). **(B)** Current-voltage relationships corresponding to **(A)**. **(C)** Gating strategy to discriminate between live and dead cells as well as apoptotic cells. **(D)** + **(E)** Distribution of dead and apoptotic cells after 2.5 h of treatment with pH 4.5 (n = 3). Statistical significance has been determined by ordinary one-way ANOVA.

TMEM206 was reported to contribute to acid-induced cell death. Therefore, we aimed to elucidate the contribution of TMEM206 to acid-induced cell death in colorectal cancer cells. First, we generated TMEM206 knockout cells by CRISPR/Cas9. None of the six obtained knockout clones shows mRNA expression for the targeted exon (Supplementary Figure S1) or acid-induced currents (Figures 2A, B) demonstrating that in HCT116 cells PAORAC/ASOR is exclusively mediated by TMEM206. We tested different commercially available antibodies against TMEM206 (Abcam ab99055, Alomone Labs ACL-031, Sigma-Aldrich ZRB1745, Novus Biologicals NBP2-85944, NBP1-88325 and NBP1-70729), but could not detect TMEM206 protein in HCT116 cells on Western Blots. Therefore, we cannot exclude expression of truncated TMEM206 protein. To test the contribution of TMEM206 to acid-induced cell death, we treated HCT116 and HCT116 TMEM206 KO cells with pH 4.5 DMEM medium for 2.5 h. Cell death was determined by SYTOX AADvanced Dead Cell staining and the mode of cell death by caspase 3/7 activity (Figure 2C). Cell death rates after low pH treatment were higher in TMEM206 KO clone 1 to 4 with 86%, 79%, 88%, and 82%, respectively *versus* 75% in parental HCT116 cells (Figure 2D). KO clones 5 and 6 showed lower cell death rates with 60% and 59% respectively. Caspase 3/7 activity rate ranged from 2.6% in clone 4 to 6.8% in clone 5, while in parental cells it was 6.6% (Figure 2E). None of the clones showed a significant change in cell death or apoptosis. In conclusion, in HCT116 cells, TMEM206 does not contribute to acid-induced cell death.

### 3.3 Compounds with structural similarity to flufenamic acid and niflumic acid inhibit TMEM206-mediated currents

**FIGURE 3:**
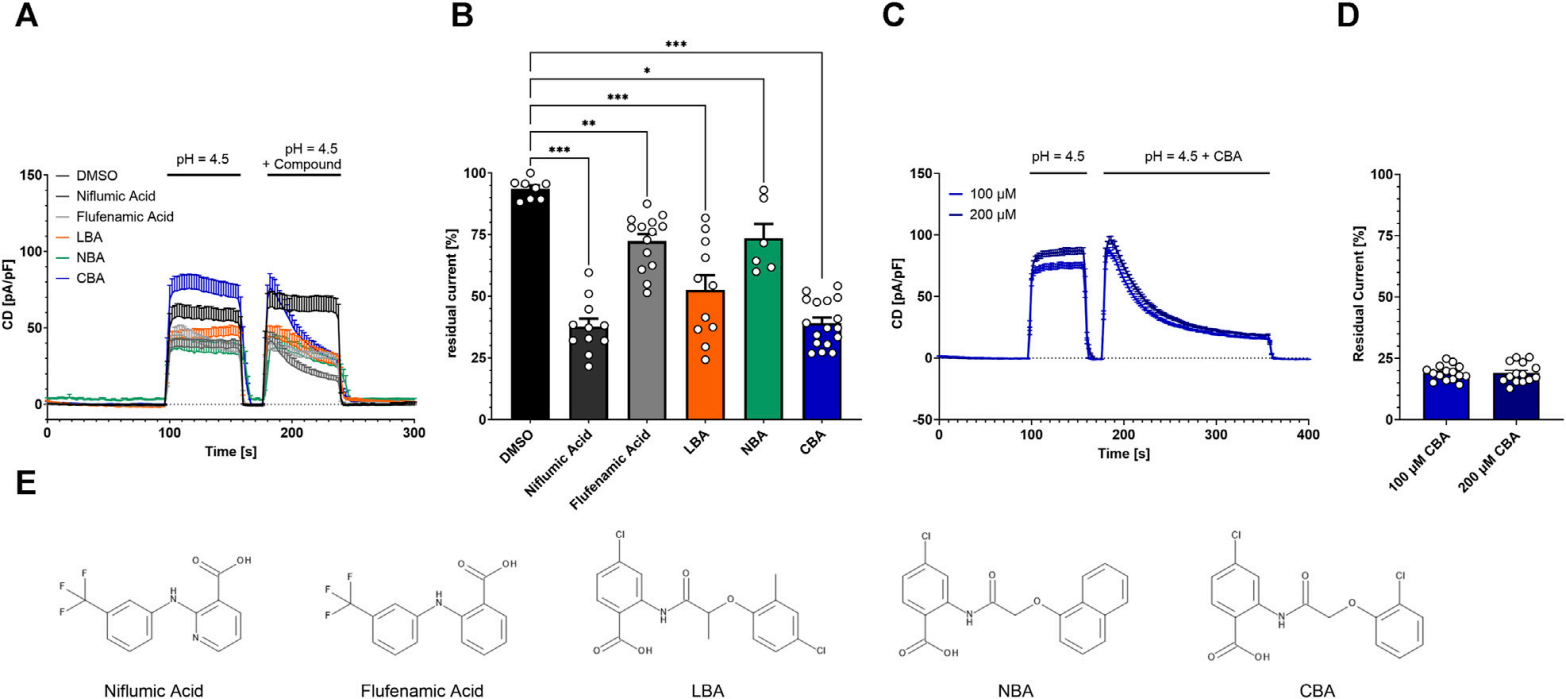
Inhibition of TMEM206 by different compounds. **(A)** Current densities when compounds are applied as indicated (Control n = 8, Niflumic Acid n = 11, Flufenamic Acid n = 14, LBA n = 11, NBA n = 6, CBA n = 17). **(B)** Inhibitory potential analyzed as residual current (current density at 236 s divided by current density at 182 s, extracted from **(A)**). Statistical significance has been determined by an unpaired t-test. **(C)** Prolonged application protocol (100 μM, n = 14; 200 μM, n = 14). **(D)** Residual currents (current density at 356 s divided by current density at 184 s) extracted from **(C)**. **(E)** Chemical structures of the used compounds in **(A)** to **(D)**; LBA = 4-chloro-2-(2-(4-chloro-2-methylphenoxy)propanamido)benzoic acid, NBA = 4-chloro-2-(1-naphthyloxyacetamido)benzoic acid, CBA = 4-chloro-2-(2-chlorophenoxy)acetamido)benzoic acid.

Both, flufenamic acid and niflumic acid inhibit PAORAC/ASOR (Lambert and Oberwinkler, 2005; Wang et al., 2007). To test if TMEM206 is inhibited by structurally similar compounds that arose from a LBVS performed earlier (Delalande et al., 2019), we applied them at a concentration of 100 µM and determined TMEM206 current inhibition. First, we activated the current by application of bath solution with pH 4.5, briefly deactivated by switching back to bath solution with pH 7.2 and activated again with pH 4.5 with the added compound or DMSO as solvent control (Figure 3A). The application of DMSO alone results in a reduction of the residual current to 93% (Figure 3B). The already-known TMEM206 blocker niflumic acid reduced residual currents to 38%. Flufenamic acid only slightly inhibited TMEM206 currents by 28%. The compounds LBA, NBA, and CBA, reduced residual currents to 53%, 74%, and 39%, respectively. Since all compounds fail to fully inhibit TMEM206 currents, we wanted to test if a longer application time leads to more effective current inhibition. Therefore, we tripled the application time from 60 s to 180 s (Figure 3C). Longer application of CBA results in lower currents (Figures 3C, D), 19% *versus* 39% for the short application. Notably, the reversal potential was shifted to more positive values upon application of 100 µM CBA pointing to a change in ion channel selectivity (Supplementary Figure S2). We also tested if a higher CBA concentration results in more efficient current inhibition. Doubling CBA concentration fails to further increase current inhibition, demonstrating that the inhibition to 19% is the maximal inhibition with CBA (Figure 3D).

### 3.4 CBA inhibits TMEM206 in a dose-dependent manner

**FIGURE 4:**
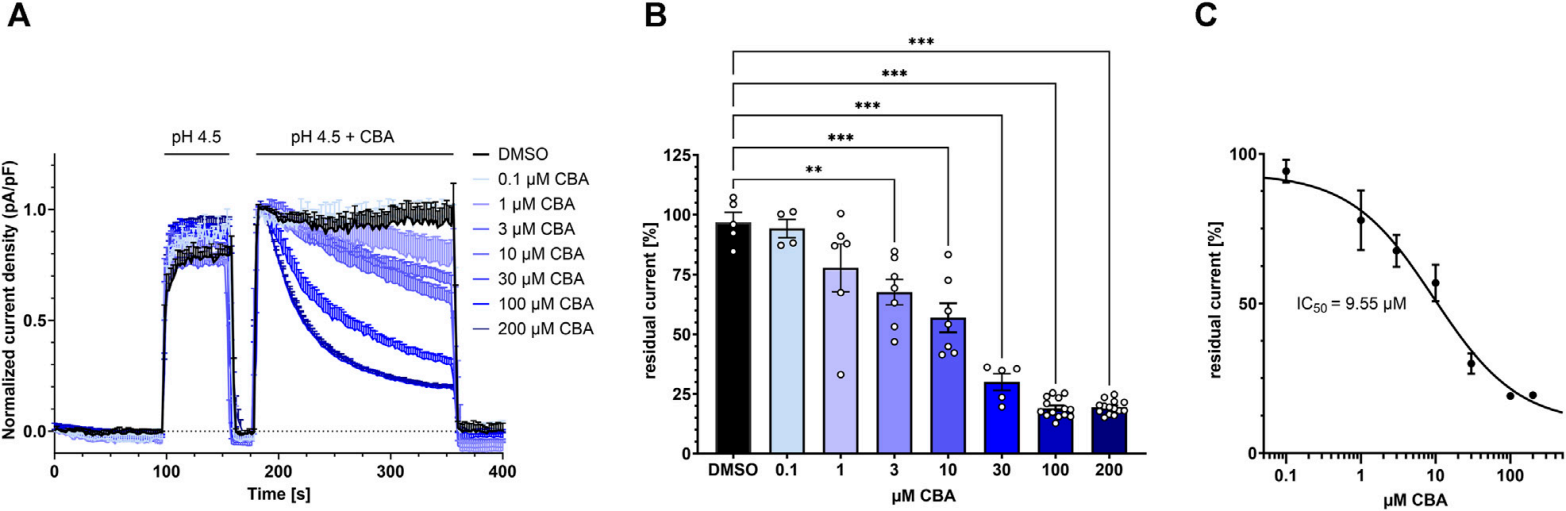
Dose-response to CBA treatment. **(A)** TMEM206 currents plotted when various concentrations of CBA were applied (control n = 5; 0.1 µM, n = 4; 1 μM, n = 6; 3 μM, n = 7; 10 μM, n = 7; 30 μM, n = 5; 100 μM, n = 14; 200 μM, n = 14). Current density has been normalized to the current density at t = 186 s. **(B)** Residual currents (current density at 356 s divided by current density at 184 s) extracted from **(A)** plotted against CBA concentration **(C)** Fitted dose response curve with means extracted from **(B)**. Calculated IC_50_ is 9.55 µM. Statistical significance has been determined by ordinary one-way ANOVA.

To test for the CBA half maximal inhibitory concentration (IC_50_) for TMEM206, we applied different concentrations ranging from 0.1 µM to 200 µM CBA and determined residual current densities. We found that CBA inhibits TMEM206 currents in a dose-dependent manner (Figure 4A, B). We fitted a non-linear Hill equation to calculate the IC_50_ for CBA, which is 9.55 µM (Figure 4C).

### 3.5 Analysis of CBA inhibition at pH 6.0

The tumor microenvironment and inflammation are often characterized by tissue acidosis, and pH values drop below pH 6.0 (Justus et al., 2013; Riemann et al., 2015). Additionally, as already shown by Sato-Numata et al., TMEM206 activation threshold shifts to pH 6.0 at 37°C, while it is inactive at physiological pH. Thus, CBA needs to be a potent inhibitor at pH 6.0 to study TMEM206 in cancer and inflammatory processes.

**FIGURE 5:**
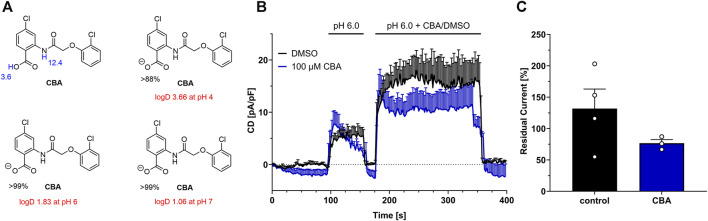
CBA at pH 6. **(A)** Calculated pK_a_ values (blue), and major microspecies (in %) and logD values (red) for CBA at different pH values (using MarvinSketch v19.27.0). **(B)** TMEM206 current inhibition by CBA at pH 6.0. (DMSO, n = 4; CBA, n = 3) **(C)** Residual currents (current density at 356 s divided by current density at 184 s) extracted from **(B)**.

Calculations show that at pH 6, CBA is 99% dissociated to its anionic form CBA_A-_ and H^+^, while at pH 4 only 88% of CBA is dissociated (Figure 5A). In addition, logD values indicate that CBA is a lipophilic substance with a more lipophilic character in low pH pointing to reduced solubility. Indeed, at pH 4.5, we observed some turbidity at higher CBA concentrations (≥30 µM), that might arise from precipitation.

We next tested if CBA blocks TMEM206 currents at pH 6.0, as our calculations show that at pH 6.0 CBA is 99% dissociated to CBA_A-_, while the logD value is only half of the logD value at pH 4.0. Since TMEM206 is only slightly active at pH 6 at room temperature, we transiently overexpressed TMEM206 in TMEM206 knockout HCT116 cells to obtain higher current densities (Figure 5B). Under these conditions, 100 µM CBA only slightly inhibits TMEM206 currents. In contrast to a mean residual current of ∼19% at pH 4.5 when 100 µM CBA were applied, residual current is at ∼77% at pH 6 with the same CBA concentration. Interestingly, DMSO application increased TMEM206 currents at pH 6.0 by 32% (Figure 5C). In conclusion, at pH 6, CBA insufficiently blocks TMEM206 and is not feasible for cellular assays and can only be used in mechanistic studies at low pH.

## 4 Discussion

• The molecular component of PAORAC/ASOR has just recently been described as the transmembrane protein TMEM206. With newer studies showing a contribution of TMEM206 in the prevention of endosomal hyperacidification and in macropinosome shrinkage, TMEM206 was initially reported to contribute to acid-induced cell death as a plasma membrane protein. We here tested if TMEM206 contributes to acid-induced cell death in HCT116 colorectal cancer cells. We first generated TMEM206 KO cells to elucidate the role of TMEM206 in acid-induced cell death in HCT116 colorectal cancer cells. We determined the rate of cell death by SYTOX AADvanced Dead Cell staining and additionally caspase 3/7 activity to test the mode of cell death. Four out of six knockout clones showed slightly increased or similar rates of cell death compared to parental HCT116 cells. KO clones 5 and 6 showed a slight reduction in cell death. In other cellular systems, both knockout or knockdown of TMEM206 led to a significant reduction in acid-induced cell death in HEK293 cells and rat cortical neurons, respectively (Ullrich et al., 2019; Osei-Owusu et al., 2020). From that we conclude that TMEM206 does not contribute to acid-induced cell death in HCT116 cells. This might be explained by the origin of HCT116 cells. These cells derive from colonic epithelium that is exposed to low pH under physiological conditions and might therefore have compensatory mechanisms that protect them from an acidic environment. In addition, the low percentage of caspase-3/7 activity points towards necrotic cell death upon acid exposure which is in line with the literature (Wang et al., 2007; Sato-Numata et al., 2014).
• The physiological role of TMEM206 was well researched in recent publications, yet the pharmacology of the channel remains not fully understood. We here show that the small molecule CBA is an inhibitor of TMEM206-mediated currents. CBA inhibits TMEM206 with a decent potency at an IC_50_ of 9.55 µM (Figure 4C). Although the IC_50_ of CBA is higher than those of DIDS (2.9 μM, Lambert and Oberwinkler, 2005; Capurro et al., 2015) and PS (5.1 μM, Drews et al., 2014), CBA has a higher potency than flufenamic acid and similar potency to niflumic acid (Figure 2B). Those compounds show structural similarity to CBA but are non-specific inhibitors of TMEM206 (Liantonio et al., 2007; Guinamard et al., 2013).
• Our original aim was to test whether CBA could prevent acid-induced cell death in HCT116 colorectal cancer cells. However, we did not find a role for TMEM206 in acid-induced cell death. In the future, CBA may be tested for its potency to reduce TMEM206 mediated acid-induced cell death in other cellular systems.
• One drawback of CBA-mediated inhibition is that currents are never fully blocked. For the short application protocol, residual currents remain at 39%, while a prolonged application time leads to a further reduction to 19% (Figure 3A, C).
• Interestingly, Mihaljevic et al. reported that PIP_2_ inhibits TMEM206 by stabilizing the channel in a desensitized-like conformation at low pH (pH < 5). Furthermore PIP_2_-mediated current inhibition was incomplete at pH 5.0 (Mihaljević et al., 2023). CBA barely inhibits TMEM206-mediated currents at pH 6.0 when TMEM206 is transiently overexpressed in TMEM206 KO HCT116 cells (Figure 5B, C). This might indicate that CBA, analogous to PIP_2_ is inhibiting TMEM206 by stabilizing its desensitized conformation at low pH. Another explanation of the observed effect at pH 6.0 is that only CBA in its protonated form, that is absent at pH 6.0, inhibits the TMEM206 channel. Yet, further research is needed to determine the mechanism of CBA-mediated TMEM206 inhibition.
• CBA has already been reported as a potent inhibitor of transient receptor potential melastatin 4 (TRPM4) channels (Ozhathil et al., 2018; Borgström et al., 2021a; Stokłosa et al., 2021) that are also expressed in colorectal cancer cells (Kappel et al., 2019; 2022; Stokłosa et al., 2022) but TRPM4-specific currents are blocked at negative physiological potentials in low pH (Supplementary Figure S3). This makes CBA a selective inhibitor of TMEM206 at low pH that can be used with certain limitations: 1) at room temperature and pH 6.0 CBA is barely inhibiting TMEM206 currents which restricts CBA use for *in vitro* assays. 2) low solubility of CBA at pH 4.5 might limit the effectiveness of inhibition and the calculated IC_50_ value should be taken with caution. Nevertheless, CBA is a good pharmacological tool to study TMEM206 function in the plasma membrane that in addition serves as a potent scaffold for the development of more potent and highly specific TMEM206 inhibitors.

## 5 Scope Statement

In neurons, HeLa, and HEK293 cells, PAORAC/ASOR (TMEM206) is a critical mediator of acid-induced cell death. In our study, we find that TMEM206 is expressed in all tested colorectal cells. In HCT116 colorectal cancer cells, PAORAC/ASOR is exclusively mediated by TMEM206. Notably, in HCT116 cells, TMEM206 is not contributing to acid-induced cell death. Our findings question the role of TMEM206 as a mechanistic key player in acid-induced cell death in cells derived from colorectal tissues and colorectal cancer cells. Additionally, our study introduces 4-chloro-2-(2-chlorophenoxy)acetamido) benzoic acid (CBA) as small molecule inhibitor of TMEM206 at low pH. In contrast to already known inhibitors, CBA specifically inhibits TMEM206 at pH 4.5 and therefore can serve as a good pharmacological tool in mechanistic studies of TMEM206. In the future, CBA scaffold may be used for the screening and identification of more potent and specific TMEM206 inhibitors.

Our findings lie within the interest of the readership of Frontiers in Pharmacology with a focus on the pharmacology of ion channels and ion channel (patho)physiology.

## Data Availability

The original contributions presented in the study are included in the article/Supplementary Material or are available in the online data depository (https://zenodo.org/doi/10.5281/zenodo.10678875).
