# Supplementary material for: CBA (4-chloro-2-(2-chlorophenoxy)acetamido) benzoic acid) inhibits TMEM206 mediated currents and TMEM206 does not contribute to acid-induced cell death in colorectal cancer cells
Source: Front Pharmacol. 2024 Mar 7;15:1369513. doi: 10.3389/fphar.2024.1369513 (PMC10955468; doi:10.3389/fphar.2024.1369513)
Supplement: Supplementary file 1 [file DataSheet1.docx]

Supplementary Material


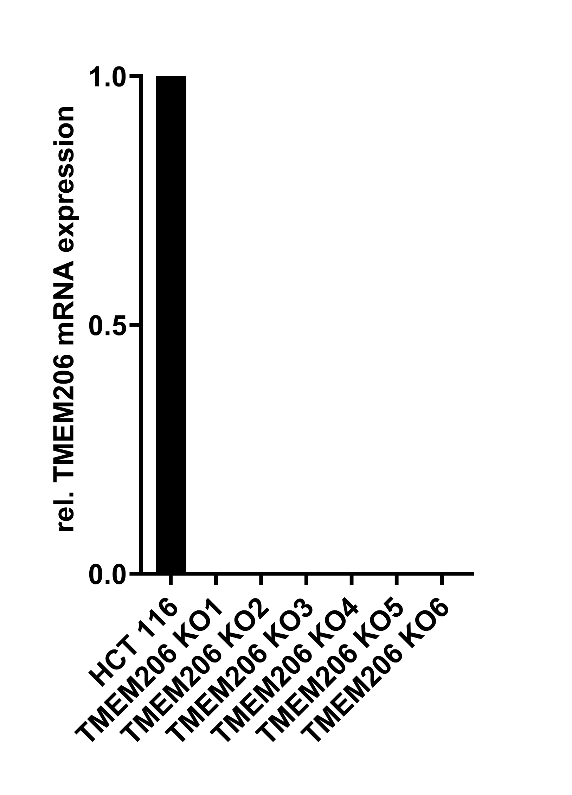


Supplementary Figure 1: quantitative PCR of HCT 116 TMEM206 KO clones.


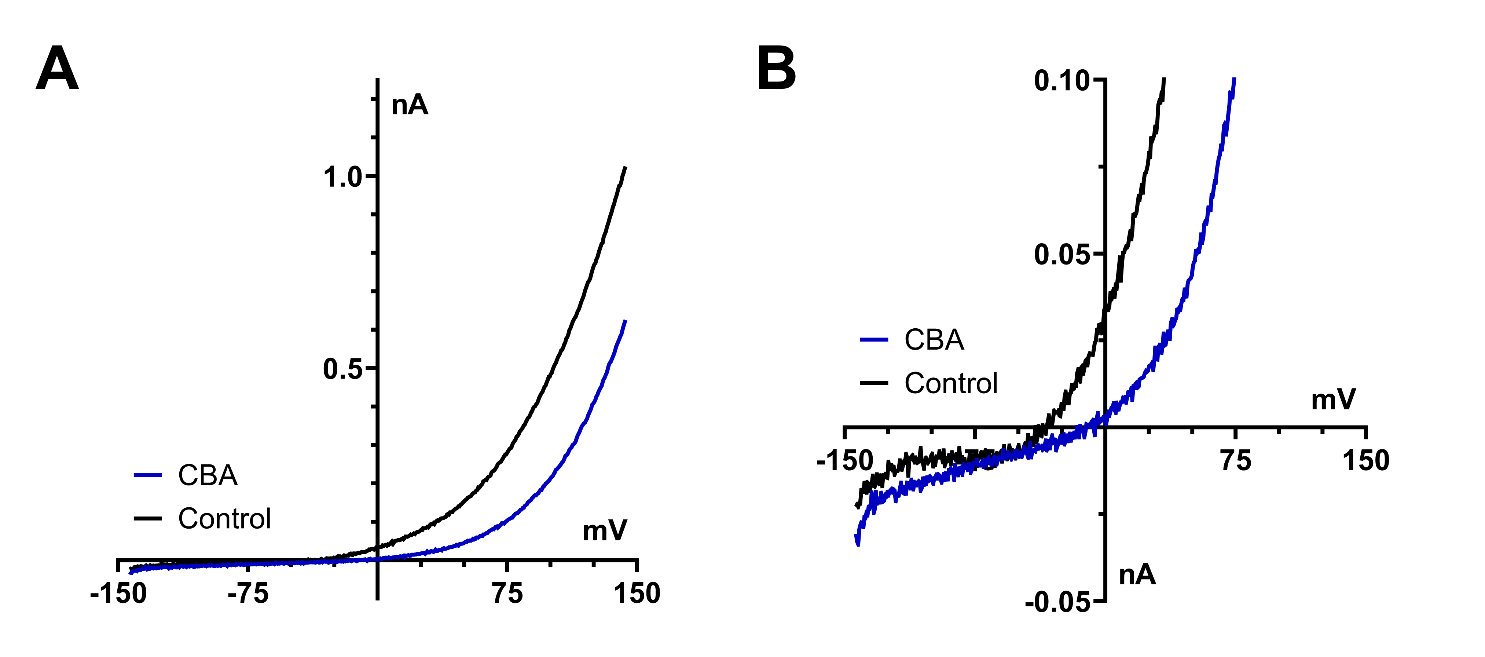


**Supplementary Figure 2: (A) Current-voltage relationships of HCT116 when 100 µM CBA was applied vs control. (B) Magnification from (A).**

**

**

Supplementary Figure 3: TRPM4 currents at pH 4.5. (A) Current density development over time when cells were exposed to pH 4.5 bath solution as indicated (n = 6). (B) Current-voltage relationships extracted from (A).
